# Obesity-associated gene mutations across cancer types: a pan-cancer analysis of TCGA data

**DOI:** 10.1038/s44276-026-00214-0

**Published:** 2026-03-23

**Authors:** Gaetana Porcelli, Rosario Nicola Brancaccio, Sebastiano Di Bella, Caterina D’Accardo, Francesco Orilio, Vincenzo Davide Pantina, Chiara Modica, Francesco Verona, Paola Bianca, Cesare Morgante, Simone Di Franco, Miriam Gaggianesi, Veronica Veschi, Giorgio Stassi, Alice Turdo, Matilde Todaro

**Affiliations:** 1 Department of Health Promotion Sciences, Internal Medicine and Medical Specialties (PROMISE), University of Palermo, Palermo, Italy; 2 Department of Precision Medicine in Medical, Surgical and Critical Care (MePreCC), University of Palermo, Palermo, Italy; 3 Catholic University of the Sacred Heart, Rome, Italy; 4 Department of Molecular Medicine, University of Rome La Sapienza, Rome, Italy; 5 IRCCS SYNLAB SDN, Naples, Italy; 6 Azienda Ospedaliera Universitaria Policlinico “Paolo Giaccone” (AOUP), Palermo, Italy

## Abstract

**Background:**

Obesity is a recognized risk factor for numerous cancers. Although several biological mechanisms have been proposed to explain obesity-associated carcinogenesis, the extent to which excess adiposity influences tumor genomic profiles remains incompletely understood. In particular, whether obesity-related selective pressures shape cancer-specific mutational landscapes is still underexplored.

**Methods:**

A pan-cancer analysis of non-synonymous somatic mutations across 14 tumor types using data from The Cancer Genome Atlas (TCGA) has been conducted. Body mass index (BMI) at diagnosis was analyzed as a continuous variable. Associations between gene mutations and BMI were assessed using logistic regression models adjusted for age, sex, and tumor mutational burden, with false discovery rate correction. Genes were prioritized using a two-step ranking strategy based on mutation frequency and regression strength. Functional inactivation, exon-level mutation distribution, and Gene Ontology enrichment analyses were performed for significantly BMI-associated genes.

**Results:**

In particular, bladder urothelial cancer (BLCA) resulted as the most frequently mutated neoplasia in association with higher body mass index. Among Eighty-six genes significantly associated with BMI in BLCA, a prioritized set of ten genes *(BRCA2, DNAH9, GRIA4, PLXNA4, UNC13C, FCGBP, SF3B1, ELP1, NES, TRERF1)* has been selected for further analyses. Overweight and obese patients exhibited distinct BMI-specific exon-level mutational patterns and concurrent deleterious mutations across multiple candidate genes. Functional inactivation analysis suggested loss-of-function mechanisms in most top-ranked genes, while Gene Ontology (GO) analysis highlighted deregulation of extracellular matrix–related pathways.

**Discussion:**

These findings support a role for obesity in shaping the genomic landscape of tumors, highlighting the importance of integrating clinical parameters such as BMI into genomic studies to determine the potential impact of obesity on tumor evolution, heterogeneity, and treatment response.

## Background

The global prevalence of obesity has reached epidemic proportions, posing a significant threat to public health worldwide [1]. Obesity is the second most common cause of preventable death after smoking and is defined by the World Health Organization as a body mass index (BMI) of 30 kg/m² or higher, while this limit is set at 25 kg/m² for Asian and South Asian populations [2]. Its rapid rise carries profound socioeconomic consequences, involving direct and indirect healthcare costs for obesity-related comorbidities, such as diabetes, cardiovascular diseases, and impaired immunity [3]. Even more alarming is the well-established link between obesity and an increased risk of several types of cancer, including endometrial, breast, ovarian, prostate, liver, bladder, kidney, colorectal, and oesophageal cancer [4–7]. Although various mechanisms have been proposed to explain the association between obesity and tumor development, the precise molecular pathophysiology driving obesity-associated carcinogenesis remains incompletely characterized.

Nonetheless, obesity-related factors such as chronic inflammation, hormonal dysregulation, and aberrant growth factor signaling are thought to contribute to increased cancer risk by promoting cellular proliferation, angiogenesis, and the evasion of apoptosis [8, 9]. Recent studies suggest that obesity may promote genomic instability through oxidative DNA damage caused by reactive oxygen species [10]. In this context, we hypothesize that the resulting selective pressures might lead to the accumulation of DNA mutations and influence tumor profile, an area still underexplored in the literature [11].

## Methods

### Data acquisition and pre-processing

Non-synonymous mutation and associated clinical data for all projects within The Cancer Genome Atlas (TCGA) were downloaded via the GDC (Genomic Data Commons) and processed using the TCGAbiolinks (v2.34.1) and maftools (v2.22.0) packages in R (v4.3.1). For each cancer type with available weight and height data, the BMI at diagnosis was calculated, categorizing patients into BMI < 25 kg/m² (underweight/normal weight) and BMI ≥ 25 kg/m² (overweight/obese) and also into BMI ≥ 30 kg/m² (obese) and 18.5 kg/m² ≤BMI < 25 kg/m² (normal weight). Fourteen cancer types satisfied these requirements (BLCA (*n* = 462), CESC (*n* = 145), CHOL (*n* = 44), COAD (*n* = 233), DLBC (*n* = 33), ESCA (*n* = 175), KIRP (*n* = 213), LIHC (*n* = 130), SKCM (*n* = 250), THYM (*n* = 100), READ (*n* = 74), UCS (*n* = 52), UCEC (*n* = 517), UVM (*n* = 53)). For each gene, a binary matrix (0/1) indicating the absence or presence of non-synonymous mutations (Missense, Nonsense, Frame Shift, In Frame, Splice Site) per patient was generated. Patients with missing or incomplete BMI data were excluded from the analysis.

### Regression model for mutation-BMI association

To identify genes whose mutations were associated with BMI, logistic regression was performed for each gene across all cancer types included in the pan-cancer analysis, with mutation status as the dependent variable and continuous BMI as the primary predictor. *p*-values were corrected for multiple testing using the Benjamini-Hochberg (BH) method to control the False Discovery Rate (FDR). An adjusted *p*-value (p.adj) < 0.05 was considered statistically significant. Concurrently, for each gene and cancer type, the frequency of non-synonymous mutations within the BMI categories (normal weight/underweight and overweight/obese) was calculated.

Additionally, we performed a logistic regression including continuous BMI along with covariates (age at diagnosis, gender, and tumor mutational burden (TMB)) to correct for potential confounders.

### Statistical analysis and gene ranking

Genes were ranked using a two-tiered approach, based on logistic regression results and mutation frequency:

Initial filtering: Genes were initially filtered to include only those with an adjusted *p*-value less than 0.05 (p.adj < 0.05), indicating a statistically significant association with BMI.

Primary ranking: These significant genes were then primarily ranked in descending order based on the absolute number of patients harboring the mutation within the BMI ≥ 25 category (N. patients mutated). This criterion prioritized genes most prevalent in the overweight/obese group.

Secondary ranking: To further refine the ranking among genes with a similar number of mutated patients, a secondary sort was performed based on the magnitude of the regression coefficient (absolute value of the Estimate), in descending order. This allowed for prioritizing genes that, in addition to being prevalent, showed a stronger estimated effect associated with BMI.

The *p*-value was calculated using a Wald test on the logistic regression coefficient for BMI, testing if it significantly differs from zero. The raw *p*-values were then corrected for multiple testing using the BH method, with significance set at p.adj < 0.05.

### Functional inactivation analysis

For genes identified as significantly associated with BMI in TCGA-BLCA cohort, we performed functional inactivation analysis to assess potential loss-of-function mechanisms. This analysis included:

Truncating mutation burden: Calculation of the percentage of nonsense, frameshift, and splice site mutations for each gene.

Biallelic inactivation assessment: Identification of patients with ≥ 2 mutations in the same gene, suggesting potential biallelic inactivation.

Recurrent hotspot analysis: Detection of genomic positions mutated in ≥ 2 patients.

Evidence classification: Genes were classified as having “STRONG EVIDENCE”, “MODERATE EVIDENCE”, or “NO EVIDENCE” of functional inactivation based on biallelic patterns, truncating mutation rates, and hotspot recurrence.

### Gene Ontology enrichment analysis

Gene Ontology (GO) enrichment analysis was performed using ClueGO (v2.5.9) within Cytoscape (v3.9.1) on the 86 bladder cancer susceptibility genes significantly associated with BMI (p.adj < 0.05). Enrichment was tested against the GO Biological Process, Cellular Component, and Molecular Function databases (EBI-UniProt-GOA-ACAP-ARAP release 25.05.2022), KEGG, Reactome Pathways, and WikiPathways using a two-sided hypergeometric test with BH correction (FDR < 0.05).

### Software and packages

All analyses were performed in R version 4.3.1 using the following key packages: TCGAbiolinks (v2.34.1), maftools (v2.22.0), dplyr (v1.1.2), ggplot2 (v3.4.2), and related dependencies.

## Results

Obesity-induced oxidative stress has been linked to genomic instability through reactive oxygen species. Therefore, we hypothesized that this phenomenon may generate selective pressures that drive the accumulation of DNA mutations and shape tumor profiles in obesity-associated cancers. To investigate whether obesity influences mutational burden and tumor features, we performed a comprehensive analysis of non-synonymous somatic mutations across distinct cancer types from TCGA dataset. Cohorts from 14 distinct cancer types (bladder urothelial cancer (BLCA), cervical squamous cell carcinoma and endocervical adenocarcinoma (CESC), cholangiocarcinoma (CHOL), colon adenocarcinoma (COAD), lymphoid neoplasm diffuse large B cell lymphoma (DLBC), esophageal carcinoma (ESCA), kidney renal papillary cell carcinoma (KIRP), liver hepatocellular carcinoma (LIHC), skin cutaneous melanoma (SKCM), thymoma (THYM), rectum adenocarcinoma (READ), uterine corpus endometrial carcinoma (UCS), uterine carcinosarcoma (UCEC), uveal melanoma (UVM)) containing BMI data have been analyzed to identify mutated genes significantly associated with BMI [12] (Supplementary Tables 1–3).

**Fig. 1:**
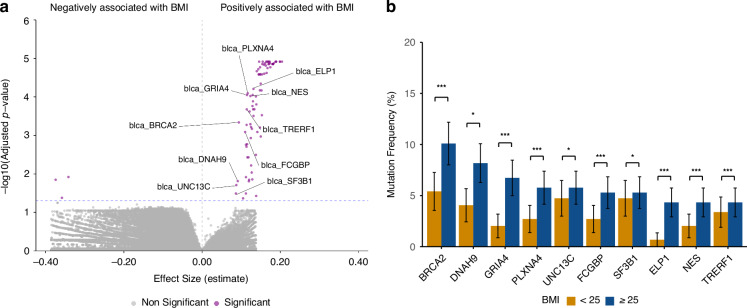
Association between BMI distribution and mutated genes across 14 cancer types in the TCGA cohort. **A** Volcano plot of the logistic regression analysis exploring the association between continuous BMI values and the presence of gene mutations across 14 cancer types within the TCGA dataset. The horizontal axis represents the regression coefficient (effect size) for BMI, while the vertical axis displays the negative logarithm base 10 of the adjusted p-value (-log10(p.adj)). The horizontal dashed line indicates the statistical significance threshold (p.adj < 0.05). Purple dots represent 86 genes harboring mutations significantly associated with BMI. Labels highlight the top ranked genes (*BRCA2*, *DNAH9*, *GRIA4*, *PLXNA4*, *UNC13C*, *FCGBP*, *SF3B1*, *ELP1*, *NES*, *TRERF1*) exhibiting a statistically significant association with BMI (p.adj < 0.05 in logistic regression analysis) and the highest mutation frequency within the BMI category ≥ 25 kg/m^2^. **B** Mutation frequencies of the top 10 ranked genes in the BMI category (yellow bar < 25 kg/m^2^ and blue bar ≥ 25 kg/m^2^) at diagnosis of TCGA patients. Data are represented as mean ± standard deviation.

Our investigation revealed a statistically significant correlation between gene mutation frequency and BMI in BLCA, as shown in Fig. 1A (*p*-value < 0.05). Detailed analysis of the BLCA cohort highlighted 86 significantly correlated genes, among which 3 genes were negatively correlated with BMI, while the remaining 83 genes showed a positive correlation (Fig. 1A and Supplementary Table 1). From the initial set of 86 genes, the top 10 candidates (*BRCA2*, *DNAH9*, *GRIA4*, *PLXNA4*, *UNC13C*, *FCGBP*, *SF3B1*, *ELP1*, *NES*, *TRERF1*) were identified by using a two-step methodology. Genes were firstly ranked in descending order according to the number of BLCA patients harbouring each mutation. Subsequently, a secondary ranking was performed based on the absolute value of the regression coefficient (Estimate), also in descending order. This strategy enabled the prioritization of genes that are both frequently mutated and display the strongest association with BMI < 25 kg/m² (including under weight BMI < 18.5 kg/m² and normal weight 18.5 kg/m² ≤BMI < 25 kg/m²) *versus* BMI ≥ 25 kg/m² (including overweigth 25 kg/m² ≤BMI < 30 kg/m² and obese BMI ≥ 30 kg/m²) (*p*-value < 0.05). Notably, after adjusting for multiple covariates (age at diagnosis, gender and tumor mutational burden), the previously identified top 10 genes persisted as significantly correlated with BMI (Fig. 1B, Supplementary Fig. 1A and Table 1).

**Table 1:** Top 10 Mutated Genes in BLCA: Combined Analysis in Patients with Elevated BMI ( ≥ 25 kg/m^2^).

Gene	Estimate	*p*.value	p.adj	BMI	N. patients mutated	Mutation frequency	Mutation frequency SD
BRCA2	0.0942	1.63E-02	0.0005	≥ 25	21	0.1009	0.0208
DNAH9	0.0878	1.00E + 00	0.0201	≥ 25	17	0.0817	0.0189
GRIA4	0.1148	2.51E-03	0.0001	≥ 25	14	0.0673	0.0173
PLXNA4	0.117	2.16E-03	0.0001	≥ 25	12	0.0576	0.0161
UNC13C	0.0865	1.7E + 00	0.0334	≥ 25	12	0.0576	0.0161
FCGBP	0.1144	6.92E-03	0.0002	≥ 25	11	0.0528	0.0155
SF3B1	0.0913	7.70E-01	0.0159	≥ 25	11	0.0528	0.0155
ELPI	0.1315	1.54E-03	0.0001	≥ 25	9	0.0432	0.0141
NES	0.1236	2.72E-03	0.0001	≥ 25	9	0.0432	0.0141
TRERFI	0.1102	3.26E-02	0.0008	≥ 25	9	0.0432	0.0141

Top 10 mutated genes, ranked primarily by number of mutated patients in the ≥ 25 kg/m^2^ BMI category and secondarily by effect size (regression coefficient) in TCGA patients. For each gene, the table displays the Gene symbol, Estimate, *p*-value, adjusted *p*-value (p.adj), the BMI category ≥ 25 kg/m^2^, the number of patients with the mutation in that category (N. patients mutated), the mutation frequency within that category, and the standard deviation of the mutation frequency. The genes listed represent those with both a significant association with BMI and a higher prevalence of mutations in the BMI ≥ 25 kg/m^2^ group.

Despite a more limited statistical power in the cohort size, comparative analysis of obese (BMI ≥ 30 kg/m²) and normal weight (18.5 kg/m² ≤BMI < 25 kg/m²) patients revealed that six out of ten candidate genes (*BRCA2, GRIA4, PLXNA4, NES, FCGBP, ELP1*) maintained top-ranking positions across both groups (Supplementary Fig. 1B). This observation strengthens the initial population-level findings and provides additional validation for the mechanistic link between obesity and BLCA pathogenesis. These findings are consistent with epidemiological data from over 500.000 participants followed for more than 20 years, which show a 1.3- to 1.5-fold increased risk of BLCA in obese as well as overweight individuals compared to those with normal weight [13]. This suggests that having a BMI above 25 kg/m² may already constitute a significant risk factor.

**Fig. 2:**
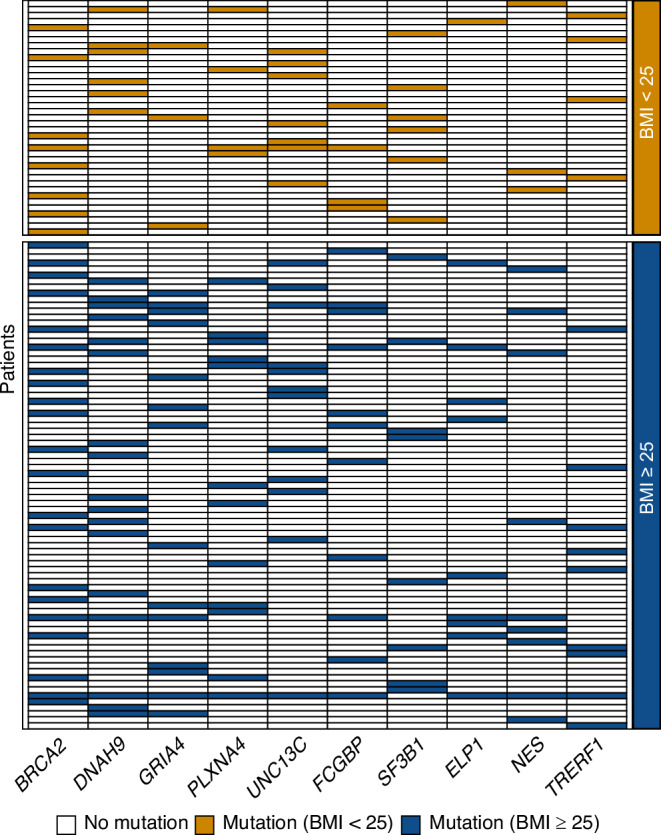
Gene mutation patterns of BMI-stratified patients. Heatmap showing non-synonymous mutations of the analyzed genes observed in each TCGA-BLCA patient grouped by BMI category.

Furthermore, while obesity did not significantly influence the magnitude of the overall increase in mutational burden (*p*-value = 0.0739) (Supplementary Fig. 1C), overweight/obese patients in our dataset frequently exhibited concurrent mutations across multiple genes, with some individuals harboring alterations in up to 9 out of the 10 genes analyzed. This suggests a substantial mutational load in this subgroup, possibly reflecting a more complex and BMI-associated genomic landscape (Fig. 2 and Supplementary Table 1).

**Fig. 3:**
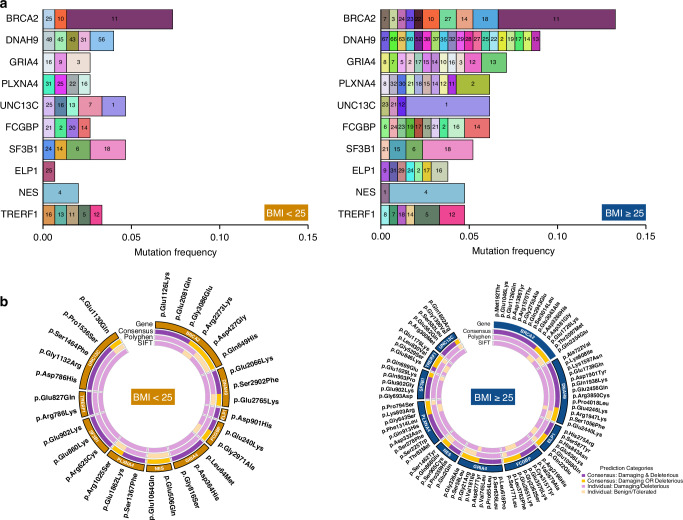
Exon mutation frequencies in ranked genes and non-synonymous mutations in patients stratified by BMI. **A** Normalized mutation frequency of top 10 ranked genes in TCGA patients, stratified by BMI category and exons. The number within bars indicates the specific mutated exon. **B** Circos plot illustrating the distribution of gene mutations exclusively present in TCGA-BLCA patients with BMI < 25 kg/m² or ≥ 25 kg/m². The outer circle shows the specific HGVSp protein sequence mutations of the indicated genes. The inferior circle represents the consensus between the bottom segments indicating in SIFT (tolerated or deleterious) or Polyphen (benign or probably/possibly damaging) pathogenicity prediction status.

For the top ten genes strongly associated with BMI, we further assessed the distribution of mutation frequencies at the exon level in BMI < 25 kg/m² and BMI ≥ 25 kg/m² patient groups (Fig. 3A).

Overall, the BMI ≥ 25 kg/m² group demonstrated a substantially higher number of mutated exons across all top ten genes with marked enrichment observed in *BRCA2*, *DNAH9*, *GRIA4*, *PLXNA4, FCGBP*, and *ELP1* (Fig. 3A and Supplementary Table 4). The normalized mutation frequency plot reveals that, although certain exons, including exon 11 of *BRCA2*, exon 4 of *NES*, exon 18 of *SF3B1*, and exon 1 of *UNC13C*, exhibit high common mutation frequencies in both BMI cohorts, each category carries distinct, non-overlapping mutations in *DNAH9*, *ELP1*, and *PLXNA4* (Fig. 3A and Supplementary Table 4). This pronounced distribution of mutations at the exon level within a specific subset of genes in TCGA-BLCA patients suggests the presence of BMI-associated mutational patterns. By inferring mutations exclusively present in one of the two categories of BMI, we highlighted a pronounced number of damaging and deleterious mutations according to SIFT and Polyphen prediction scores in ≥25 kg/m² BMI patients (Fig. 3B and Supplementary Table 4).

Thus, to better interpret the biological significance of BMI-associated mutations, we assessed whether these alterations could compromise the function of the encoded proteins. A functional inactivation analysis revealed that 24 BMI-correlated genes, and specifically 9 out of 10 of the top-ranking genes, harbor mutations that could affect their biological activity (Supplementary Table 5). In this context, GO molecular function analysis revealed a significant deregulation in the extracellular matrix (ECM) remodeling process (Supplementary Fig. 1D and Supplementary Table 6), recognized as a prognostic factor in cancer [14]. In line with previous findings, in BLCA patients, loss of collagen is associated with increased invasive behavior and reduced overall survival, as a poorly organized ECM can promote tumor initiation and facilitate tumor cell migration and invasion [15]. Overall, these results suggest that BMI-related mutational patterns may be related to adverse prognosis and tumor progression.

## Discussion

Our study highlighted the occurrence of non-synonymous mutations in ten genes significantly associated with BMI in the context of BLCA. Among the selected genes, *SF3B1* and *BRCA2* stands out as well-established drivers in the tumorigenesis of BLCA [16]. In line with *BRCA2, ELP1* plays a crucial role in DNA repair and the preservation of genomic stability. Inactivation of these genes leads to the accumulation of DNA damage and has been associated with oncogenic transformation [17, 18]. Genes such as *UNC13C*, *FCGBP*, and *NES* have been previously linked to tumor progression and stemness-related pathways in other cancer types, though their specific roles in BLCA require further investigation [19–21]. In addition, *DNAH9*, *GRIA4*, *PLXNA4*, and *TRERF1* have been reported in the literature to be involved in transcriptional regulation and in modulating the tumor microenvironment, suggesting potential, yet poorly defined, oncogenic functions [22–25].

Of note, our study offers several layers of novelty aimed at providing a more comprehensive view of BMI-associated mutational patterns in cancer.

Although we also rely on TCGA data, as in Huang et al. study [12], we implemented a uniform linear regression modeling framework across 14 cancer types to identify reproducible BMI-associated mutational changes, which has not been used in previous TCGA obesity studies. Moreover, while Tang et al. [11] analyzed only oncogenic mutations within a proprietary dataset, our study examines genome-wide patterns using publicly available data, enabling broader cross-cancer comparisons.

Building on this systematic framework, we further introduce an exon-level analysis of mutation distributions within selected genes in TCGA normal-weight versus overweight/obese patients. This finer-resolution analysis reveals distinct, non-overlapping exon-level mutational landscapes between groups, suggesting that obesity may influence not only the prevalence of mutations but also their positional distribution within genes.

This broader analytic scope also enabled us to detect a statistically significant correlation between gene mutation frequencies and BMI in BLCA. Interestingly, this result may be shaped in part by the limited availability of BMI data stratified by cancer type within the underlying datasets. Therefore, this observation underscores a central limitation across prior TCGA-based obesity studies, highlighting the necessity for larger and more comprehensive studies that incorporate clinical data, including BMI, across cancer datasets. While the mutation frequency of BLCA was assessed at a single timepoint, it is important to note that BMI fluctuates over an individual’s lifetime. Therefore, a single BMI measurement from TCGA likely reflects a combination of premorbid adiposity and potential disease-related weight changes. Thus, while the accumulation of mutations could be interpreted as evidence of a molecular “memory” of previous episodes of excessive adiposity, the inherently cross-sectional design of the study limits causal interpretation and highlights the need for longitudinal evaluation.

Although early interventions for obesity, particularly with the development of novel weight-loss pharmacotherapies, can mitigate comorbidities and enhance quality of life, patients with prolonged exposure to obesity may retain a higher frequency of obesity-associated mutations. This persistent mutational load could ultimately manifest as increased tumor heterogeneity, contributing to therapeutic resistance and modulating treatment response. Accordingly, longitudinal studies that characterize body weight trajectories over lifespan are critical for improving risk stratification and guiding personalized management strategies. These observations suggest that certain cancer patients may benefit from diagnostic and therapeutic approaches tailored to their obesity-induced mutational profiles, potentially optimizing clinical outcomes. Moreover, additional research is needed to elucidate the genetic and, more importantly, the imprinted epigenetic mechanisms by which obesity promotes cancer and whether some mechanism may be tumor specific.

## Supplementary information

Figure_s1 REV1

Supplementary Figure legends REV1

Supplementary_table_1

Supplementary_table_2

Supplementary_table_3

Supplementary_table_4

Supplementary_table_5

Supplementary_table_6

## Data Availability

All data generated or analysed during this study are included in this web link https://github.com/LabStassi/TCGA_data_analysis.

## Code availability

Code for reproducing the results showed in Fig. 1 is available at: https://github.com/LabStassi/TCGA_data_analysis.
