# Supplementary figures and images for: Obesity-associated gene mutations across cancer types: a pan-cancer analysis of TCGA data
Source: BJC Rep. 2026 Mar 23;4:13. doi: 10.1038/s44276-026-00214-0 (PMC13009199; doi:10.1038/s44276-026-00214-0)

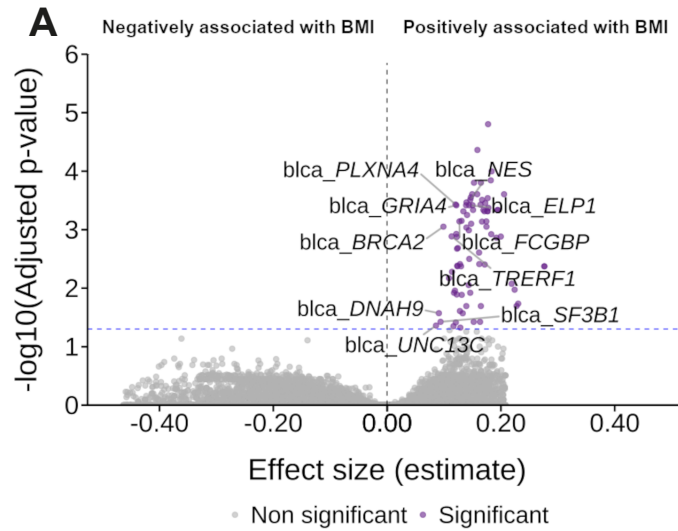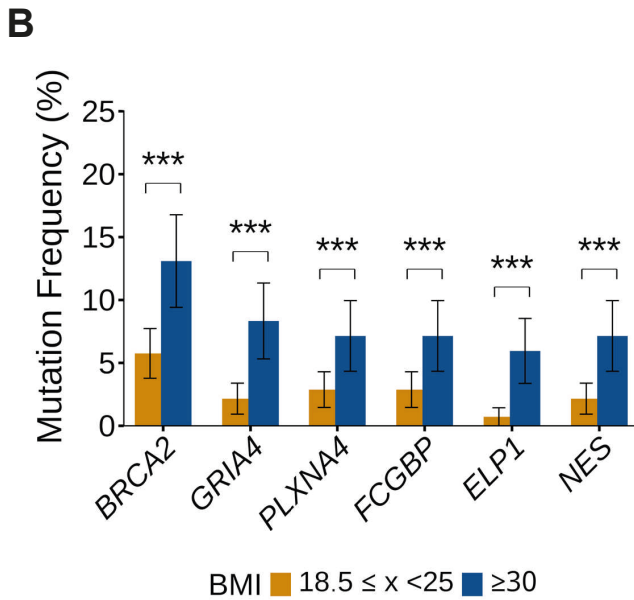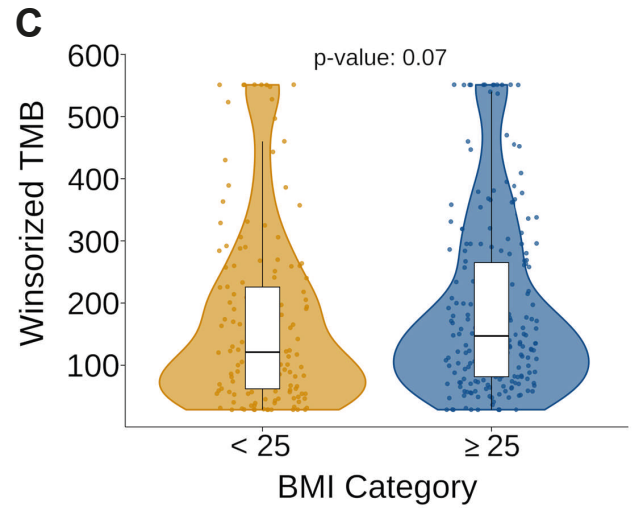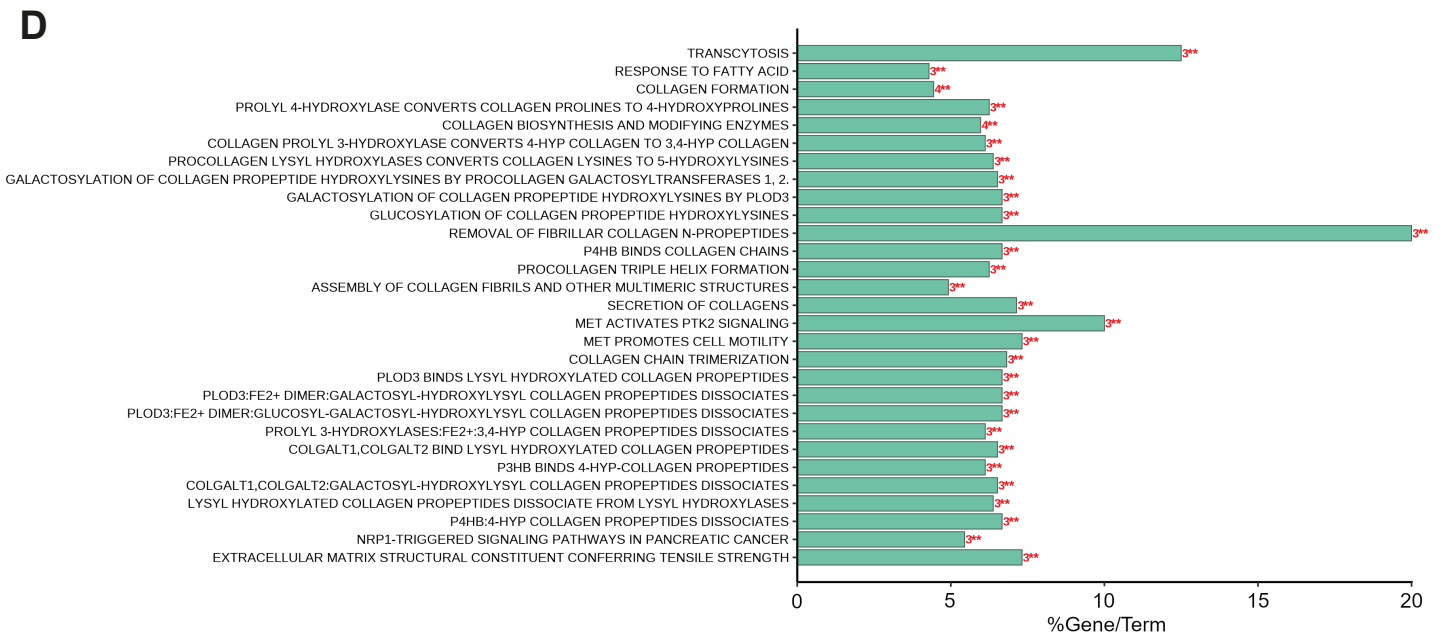

Supplement: Supplementary file 1 — Figure_s1 REV1 [file 44276_2026_214_MOESM1_ESM.pdf]
