# Supplementary material for: Obesity-associated gene mutations across cancer types: a pan-cancer analysis of TCGA data
Source: BJC Rep. 2026 Mar 23;4:13. doi: 10.1038/s44276-026-00214-0 (PMC13009199; doi:10.1038/s44276-026-00214-0)
Supplement: Supplementary file 2 — Supplementary Figure legends REV1 [file 44276_2026_214_MOESM2_ESM.docx]

**Supplementary Figure legends**

**Supplementary Figure S1. BMI-Associated Somatic Alterations Promote ECM Remodeling in BLCA patients**

**A.** Volcano plot of the logistic regression analysis exploring the association between continuous BMI values and the presence of gene mutations across 14 cancer types within the TCGA dataset adjusted for multiple covariates (age at diagnosis, gender and tumor mutational burden). The horizontal axis represents the regression coefficient (effect size) for BMI, while the vertical axis displays the negative logarithm base 10 of the adjusted p-value (-log10(p.adj)). The horizontal dashed line indicates the statistical significance threshold (p.adj < 0.05). Purple dots represent 82 genes harboring mutations significantly associated with BMI corrected from covariates. Labels highlight the top ranked genes (BRCA2, DNAH9, GRIA4, PLXNA4, UNC13C, FCGBP, SF3B1, ELP1, NES, TRERF1) exhibiting a statistically significant association with BMI (p.adj < 0.05 in logistic regression analysis) and the highest mutation frequency within the BMI category ≥ 25 kg/m^2^. **B.** Mutation frequencies of the top 10 ranked genes in the BMI category (yellow bar 18 ≤ x < 25 kg/m2 and blue bar ≥ 30 kg/m2) at diagnosis of TCGA patients. Data are represented as mean ± standard deviation. **C.** Violin plot compares the distribution of TMB between patients with BLCA classified into two BMI categories at diagnosis: BMI < 25 kg/m^2^ and BMI ≥25 kg/m^2^. The shape of the violin plot represents the density of TMB values within each group, while the overlaid points (jitter plot) show individual patient values. The boxplot within each violin indicates the median and interquartile range. **D.** Gene Ontology (GO) enrichment analysis in BLCA susceptibility genes in BMI≥25 kg/m^2^ category (p.adj < 0.05).

**Supplementary Table 1. Integrated Gene Data Across TCGA Cancer Types for Non-synonymous mutations in BMI <25 versus ≥ 25 categories.**

Comprehensive non-synonymous gene mutations across multiple cancer types from the TCGA. This table integrates data from individual cancer-specific analyses, providing the gene symbol, regression coefficient (Estimate) from the BMI association analysis (using continuous BMI as a predictor), corresponding p-value and adjusted p-value (p.adj), the BMI category exhibiting higher mutation frequency, the number of patients with the mutation in that category, the total number of patients analyzed for that gene, the mutation frequency, and the standard deviation of the mutation frequency.

**Supplementary Table 2. Integrated Gene Data Across TCGA Cancer Types for Non-synonymous mutations in normal weight (BMI≤18.5) versus obese (BMI≥30) categories.**

Integrated results of the individual cancer specific analysis, performed as in supplementary table 1, considering only the BMI categories normal (18.5≤BMI< 25) versus obese (BMI≥30).

**Supplementary Table 3. Distribution of patients by BMI category across cancer types.**

**Supplementary Table 4. Frequency of non-synonymous mutations in selected BLCA genes, stratified by BMI category and exon.**

Frequency of specific non-synonymous mutations observed for each analyzed gene in the TCGA-BLCA dataset shown in Figure 1D and 1E. Each row represents a unique combination of genomic (HGVSc), and protein (HGVSp) position ID, patient BMI category at diagnosis (< 25 kg/m^2^; ≥25 kg/m^2^), the exon number and mutation pathogenicity predictions and relative scores. The "count" column indicates the number of patients within that BMI category and exon who harbor that particular mutation, while the "patients" column lists the unique patient identifiers (Tumor_Sample_Barcode) carrying it. The "gene" column specifies the gene in which the mutation was identified.

**Supplementary Table 5. Analysis of functional inactivation in BLCA susceptibility genes stratified by BMI category.**

Analysis of functional inactivation patterns in selected BLCA susceptibility genes in the TCGA-BLCA dataset. Each row represents one gene analyzed for potential functional inactivation.

**Supplementary Table 6. Gene Ontology (GO) enrichment analysis in BLCA susceptibility genes stratified by BMI category.**

Gene Ontology (GO) enrichment analysis was performed using ClueGO (v2.5.9) within Cytoscape (v3.9.1) on the 86 BLCA susceptibility genes significantly associated with BMI (p.adj < 0.05).
